# Intranuclear inclusions in muscle biopsy can differentiate oculopharyngodistal myopathy and oculopharyngeal muscular dystrophy

**DOI:** 10.1186/s40478-022-01482-w

**Published:** 2022-12-07

**Authors:** Masashi Ogasawara, Nobuyuki Eura, Aritoshi Iida, Theerawat Kumutpongpanich, Narihiro Minami, Ikuya Nonaka, Shinichiro Hayashi, Satoru Noguchi, Ichizo Nishino

**Affiliations:** 1 Department of Neuromuscular Research, National Institute of Neuroscience, National Center of Neurology and Psychiatry (NCNP), 4-1-1 Ogawahigashi, Kodaira, Tokyo, 187-8502 Japan; 2 Medical Genome Center, NCNP, Kodaira, Tokyo, Japan; 3 Department of Pediatrics, Showa General Hospital, Kodaira, Tokyo, Japan; 4 Department of Neurology, Nara Medical University, Nara, Japan

**Keywords:** Oculopharyngodistal myopathy, LRP12, GIPC1, NOTCH2NLC, CGG repeat expansion, Oculopharyngeal muscular dystrophy, Intranuclear inclusion

## Abstract

Oculopharyngodistal myopathy (OPDM) and oculopharyngeal muscular dystrophy (OPMD) are similar and even believed to be indistinguishable in terms of their myopathological features. To address the diagnostic gap, we evaluated the muscle biopsy samples for p62 expression by immunohistochemistry and compared the occurrence and the frequency of intranuclear inclusions among the individuals with OPDM (harboring CGG repeat expansion in *LRP12* (*n* = 19), *GIPC1* (*n* = 6), or *NOTCH2NLC* (*n* = 7)), OPMD (*n* = 15), and other rimmed vacuolar myopathies. We found that myonuclei with p62-positive intra-nuclear inclusions (myo-INIs) were significantly more frequent in OPMD (11.9 ± 1.1%, range 5.9–18.6%) than in OPDM and other rimmed vacuolar myopathies (RVMs) (0.9–1.5% on average, range 0.0–2.8%, *p* < 0.0001). In contrast, INIs in non-muscle cells such as blood vessels, peripheral nerve bundles, and muscle spindles (non-muscle-INIs) were present in OPDM, but absent in OPMD. These results indicate that OPMD can be differentiated from OPDM and other RVMs by the frequent presence of myo-INIs; and in OPDM, the presence of non-muscle-INIs in muscle pathology should be a diagnostic hallmark.

**Supplementary Information:**

The online version contains supplementary material available at 10.1186/s40478-022-01482-w.

## Introduction

Oculopharyngodistal myopathy (OPDM) is characterized clinically by progressive ptosis, ophthalmoplegia, bulbar muscle involvement, and limb muscle weakness that is predominantly distal and pathologically by the presence of rimmed vacuoles [11]. Recently, OPDM was shown to be caused by a CGG repeat expansion in the noncoding region of the *Low-density lipoprotein receptor-related protein 12* (*LRP12*), *GIPC PDZ domain containing family member 1* (*GIPC1*), *Notch homolog 2 N-terminal-like protein C* (*NOTCH2NLC*), or *Rab interacting lysosomal protein like 1* (*RILPL1*) (OPDM_LRP12, OPDM_GIPC1, OPDM_NOTCH2NLC, OPDM_RILPL1) gene [2, 6, 10, 17–19]. Oculopharyngeal muscular dystrophy (OPMD) is clinicopathologically similar to OPDM. However, this muscle disease is caused by an alanine expansion mutation in the poly-adenine-binding protein nuclear 1 (*PABPN1*) gene [1]. It is also clinically characterized by oculopharyngeal muscle involvement and rimmed vacuolar pathology, but the associated limb muscle weakness is typically proximal, rather than distal [14, 16]. The histopathology of these two diseases is quite similar and indistinguishable.

Intra-nuclear inclusion (INI) is a pathological hallmark of neuronal intranuclear inclusion disease (NIID) caused by CGG repeat expansion in *NOTCH2NLC*, which is also one of the causative mutations of OPDM [10, 12, 13, 18]. Recently, it has been reported that INIs in the skin biopsy are not specific to NIID but can be seen in all three OPDM subtypes [9]. This situation naturally raises the question of how INIs are distributed in the skeletal muscle in OPDM and OPMD. In this study, we characterized the INIs in muscle biopsy specimens from patients with three types of OPDM, OPMD, NIID, as well as four other rimmed vacuolar myopathies (RVMs) to pathologically differentiate between OPDM and OPMD as the conditions are typically indistinguishable by routine muscle biopsy histochemistry.

## Materials and methods

### Subjects

All of the biopsy samples were sent to the National Center of Neurology and Psychiatry (NCNP), a referral center for muscle diseases in Japan, for diagnostic purposes; and all of the patients gave their informed consent for the use of their samples for research. We analyzed muscle biopsy samples from patients with OPDM_LRP12 (*n* = 19), OPDM_GIPC1 (*n* = 6), OPDM_NOTCH2NLC (*n* = 7), OPMD (*n* = 15), NIID (*n* = 10), inclusion body myositis (IBM) (*n* = 11), GNE myopathy (*n* = 11), inclusion body myopathy with Paget’s disease of the bone and frontotemporal dementia (IBMPFD) with valosin-containing protein gene mutation (*n* = 8), and DES-related myopathy (*n* = 7). In addition, we included six muscle biopsy samples without histopathological abnormalities as controls (Additional file 1: Table S1).

### Muscle histology

**Fig. 1:**
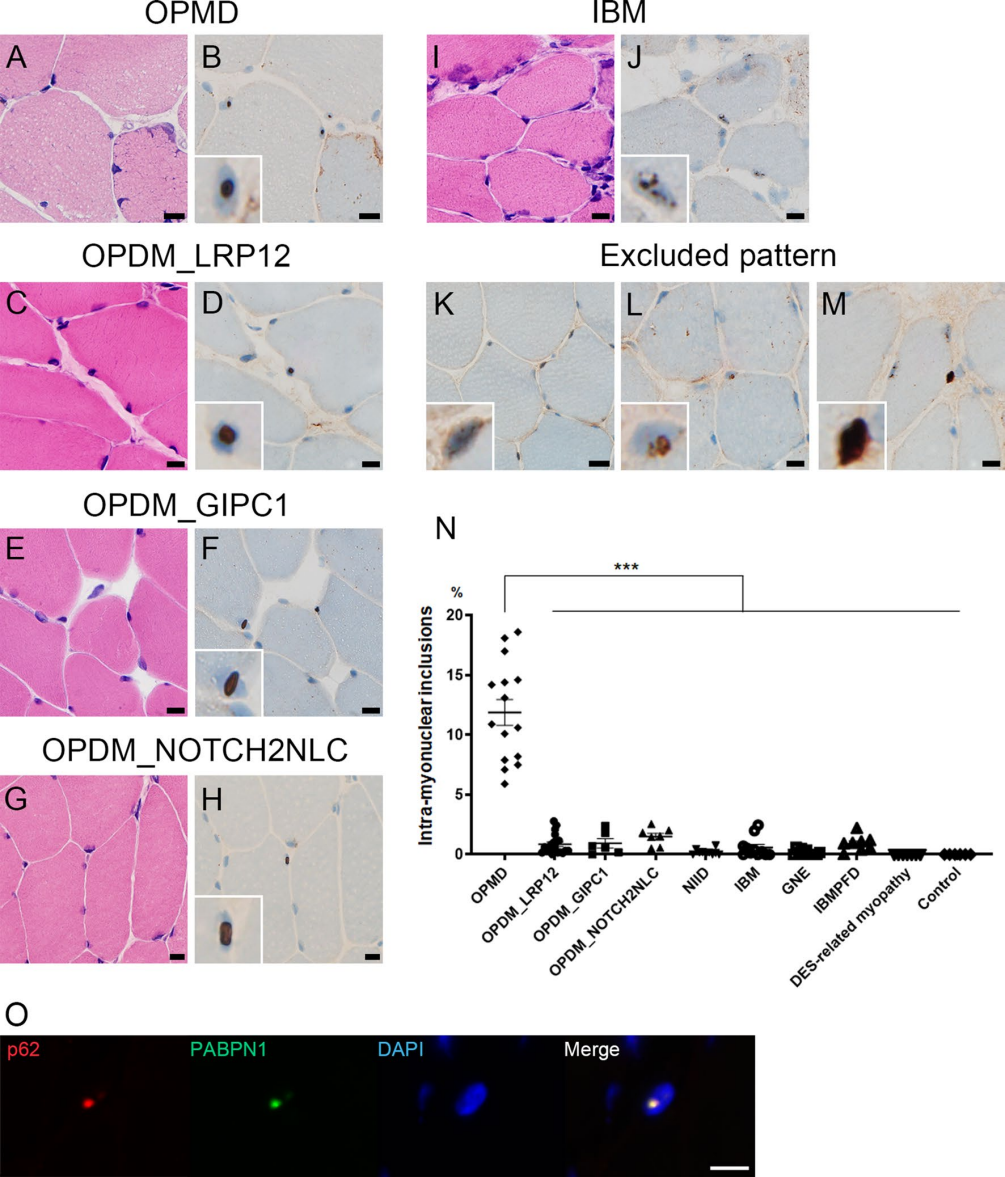
Intra-myonuclei stained by anti-p62 antibody. H&E (**a**, **c**, **e**, **g**, **i**) and p-62 antibody (**b**, **d**, **f**, **h**, **j**) staining of serial sections of the muscle sample for each disease. Excluded staining pattern of anti-p62 antibody in the control (**k**), GNE myopathy (**l**), and IBM (**m**) samples. **n** The graph shows the frequency of p62-positive intra-myonuclei in each disease. Error bars indicate mean ± SEM. ***p < 0.0001; *ns* no significance. **o** PABPN1-positive aggregates were localized in the myonuclei, similar to p62 positive myo-INIs in OPMD. **a**–**m**, **o**) All bars show 10 µm. ***OPMD shows higher frequency of p62-positive intra-myonuclei compared to OPDM_LRP12, OPDM_GIPC1, OPDM_NOTCH2NLC, NIID, IBM, GNE, IBMPFD, DES-related myopathy, and control samples (*p* < 0.0001). *H&E* hematoxylin and eosin; IBM: inclusion body myositis; *OPMD* oculopharyngeal muscular dystrophy; *OPDM* oculopharyngodistal myopathy; *NIID* neuronal intranuclear inclusion disease; *IBMPFD* inclusion body myopathy with Paget’s disease of bone and frontotemporal dementia

The immunohistochemistry assay for anti-p62/SQSTM1 (sc-28359, 1;200, Santa Cruz Biotechnology) and anti-PABPN1 (ab75855, 1:100, Abcam) was performed using the Ventana immunohistochemistry detection system (Ventana Medical Systems, Tucson, AZ, USA). Routine hematoxylin and eosin (H&E) staining was also done. Immunofluorescence analysis was also performed using the same primary antibodies of guinea pig anti-p62/SQSTM1 (sc-28359, 1;200, Santa Cruz Biotechnology), rabbit anti-PABPN1 (ab75855, 1:100, Abcam), rabbit anti-Caveolin-1 (C4490, 1:100, Sigma-Aldrich), and rat anti-Laminin α2 (sc-59854, 1:100, Santa Cruz Biotechnology) at room temperature for an hour. The sections were incubated with Alexa Fluor 647 anti-Guinea Pig (706–606-148; Jackson ImmunoResearch), 488 anti-Rabbit (A21206; Invitrogen), and 568 anti-Rat (ab175475; Abcam) secondary antibodies for an hour at room temperature, followed by mounting with DAPI (ab104139; Abcam). The images were taken using the BZ-X710 Fluorescence Microscope (Keyence, Osaka, Japan). We analyzed the frequency of anti-p62 antibody-positive intra-myonuclear inclusions (myo-INIs) in three randomly selected areas (200 × magnification) for each specimen. The myonuclei that strongly stained with anti-p62 were considered to be myo-INIs, but not the nuclei with small or > 2 dots (Fig. 1k) [5], faintly stained dots (Fig. 1l), or those that have dots which were larger than the myonuclei (Fig. 1m). We further counted the number of p62-positive nuclei in the non-muscle cells (non-muscle-INIs) contained within in the whole specimen, such as those in the blood vessels, peripheral nerve bundles, and muscle spindles, except for intrafusal muscle fibers.

### Statistical analysis

Statistical analysis was performed using GraphPad Prism version 8.4.3 for Windows (GraphPad Software, San Diego, California, USA). One-way ANOVA with Tukey's post hoc test was used to ascertain the differences of the frequency of p62-positive nuclei among OPDM_LRP12, OPDM_GIPC1, OPDM_NOTCH2NLC, OPMD, NIID, IBM, GNE myopathy, IBMPFD, DES-related myopathy, and the control samples. The difference was considered statistically significant at *p* < 0.05.

## Results

We observed 515 ± 13 (mean ± SEM; range 177–810) myonuclei per patient (*n* = 100), 122 ± 13 (range 8–785) nuclei in blood vessels (*n* = 99), 56 ± 14 (range 5–482) nuclei in Schwann cells/pericytes (*n* = 35), 35 ± 6.7 (range 4–209) nuclei in the perineurium (*n* = 35), and 24 ± 5.3 (range 4–77) nuclei in muscle spindles excluding intrafusal muscle fibers (*n* = 15). In OPMD, the myo-INIs were found in all of the patients (100%, 15/15), and involving 11.9 ± 1.1% (range 5.9–18.6%) of myonuclei. In comparison, the myo-INIs were absent in the muscle samples taken from the seven DES-related myopathy and the six controls (Fig. 1a, b, n, Table 1). In the other diseases that were evaluated in this study, the proportion of myo-INIs varied, but were found at a significantly lower frequency than in OPMD, and showed the following distribution: OPDM_LRP12 (1.0 ± 0.3%, range 0.0–2.8%, *p* < 0.0001), OPDM_GIPC1 (0.9 ± 0.4%, range 0.0–2.4%, *p* < 0.0001), OPDM_NOTCH2NLC (1.5 ± 0.3%, range 0.5–2.6%, *p* < 0.0001), NIID (0.2 ± 0.1%, range 0.0–0.8%, *p* < 0.0001), IBM (0.6 ± 0.3%, range 0.0–2.5%, *p* < 0.0001), GNE myopathy (0.2 ± 0.1%, range 0.0–0.6%, *p* < 0.0001), and IBMPFD (0.9 ± 0.2%, range 0.0–0.6%, *p* < 0.0001) (Fig. 1c–j, Table 1). Usually, single INIs were seen in the myonuclei in OPMD, three OPDM subtypes, and NIID. In contrast, the myonuclei in IBM often contained several smaller p62-positive dots (Fig. 1j). On immunohistochemistry for anti-PABPN1 in OPMD, PABPN1-positive aggregates were observed in the myonuclei, corresponding to the p62 positive myo-INIs (Fig. 1o), while PABPN1-positive aggregates were absent in the myo-INIs of the other diseases.

**Table 1:** Anti-p62 antibody-positive intra-nuclear inclusions of muscle fibers and non-muscle cells in muscle biopsy

	Muscle fiber (% of all myonuclei)	Blood vessel (% of all nuclei)	Schwann cell/Pericyte (% of all nuclei)	Perineurium (% of all nuclei)	Muscle spindle (% of all nuclei)
OPMD	11.9 ± 1.1 (*n* = 15)	0 (*n* = 15)	0 (*n* = 2)	0 (*n* = 2)	0 (*n* = 1)
OPDM_LRP12	1.0 ± 0.3 (*n* = 19)	0.7 ± 0.3 (*n* = 19)	4.0 ± 1.2 (*n* = 7)	4.1 ± 2.0 (*n* = 7)	13.2 ± 0.7 (*n* = 2)
OPDM_GIPC1	0.9 ± 0.4 (*n* = 6)	0.9 ± 0.5 (*n* = 6)	18.5 ± 8.8 (*n* = 2)	8.7 ± 2.2 (*n* = 2)	18.9 ± 6.9 (*n* = 2)
OPDM_NOTCH2NLC	1.5 ± 0.3 (*n* = 7)	8.0 ± 1.8 (*n* = 7)	13.4 ± 3.4 (*n* = 5)	15.8 ± 2.8 (*n* = 5)	16.3 ± 2.0 (*n* = 2)
NIID	0.2 ± 0.1 (*n* = 10)	7.2 ± 1.2 (*n* = 10)	8.0 ± 4.4 (*n* = 3)	21.8 ± 5.2 (*n* = 3)	9.1 (*n* = 1)
IBM	0.6 ± 0.3 (*n* = 11)	0.5 ± 0.5 (*n* = 11)	0.6 ± 0.6 (*n* = 4)	0 (*n* = 4)	0 (*n* = 3)
GNE myopathy	0.2 ± 0.1 (*n* = 11)	0 (*n* = 10)	0.8 ± 0.8 (*n* = 6)	0.5 ± 0.7 (*n* = 6)	5.5 ± 5.5 (*n* = 2)
IBMPFD	0.9 ± 0.2 (*n* = 8)	0.2 ± 0.2 (*n* = 8)	0 (*n* = 1)	2.6 (*n* = 1)	0 (*n* = 1)
DES-related myopathy	0.0 ± 0.0 (*n* = 7)	0 (*n* = 7)	0 (*n* = 3)	0 (*n* = 3)	NA
Control	0.0 ± 0.0 (*n* = 6)	0 (*n* = 6)	0 (*n* = 2)	0 (*n* = 2)	0 (*n* = 1)

Data are shown as Mean ± SEM. *OPMD* oculopharyngeal muscular dystrophy; *OPDM* oculopharyngodistal myopathy; *NIID* neuronal intranuclear inclusion disease; *IBM* inclusion body myositis; *IBMPFD* inclusion body myopathy with Paget’s disease of bone and frontotemporal dementia

**Fig. 2:**
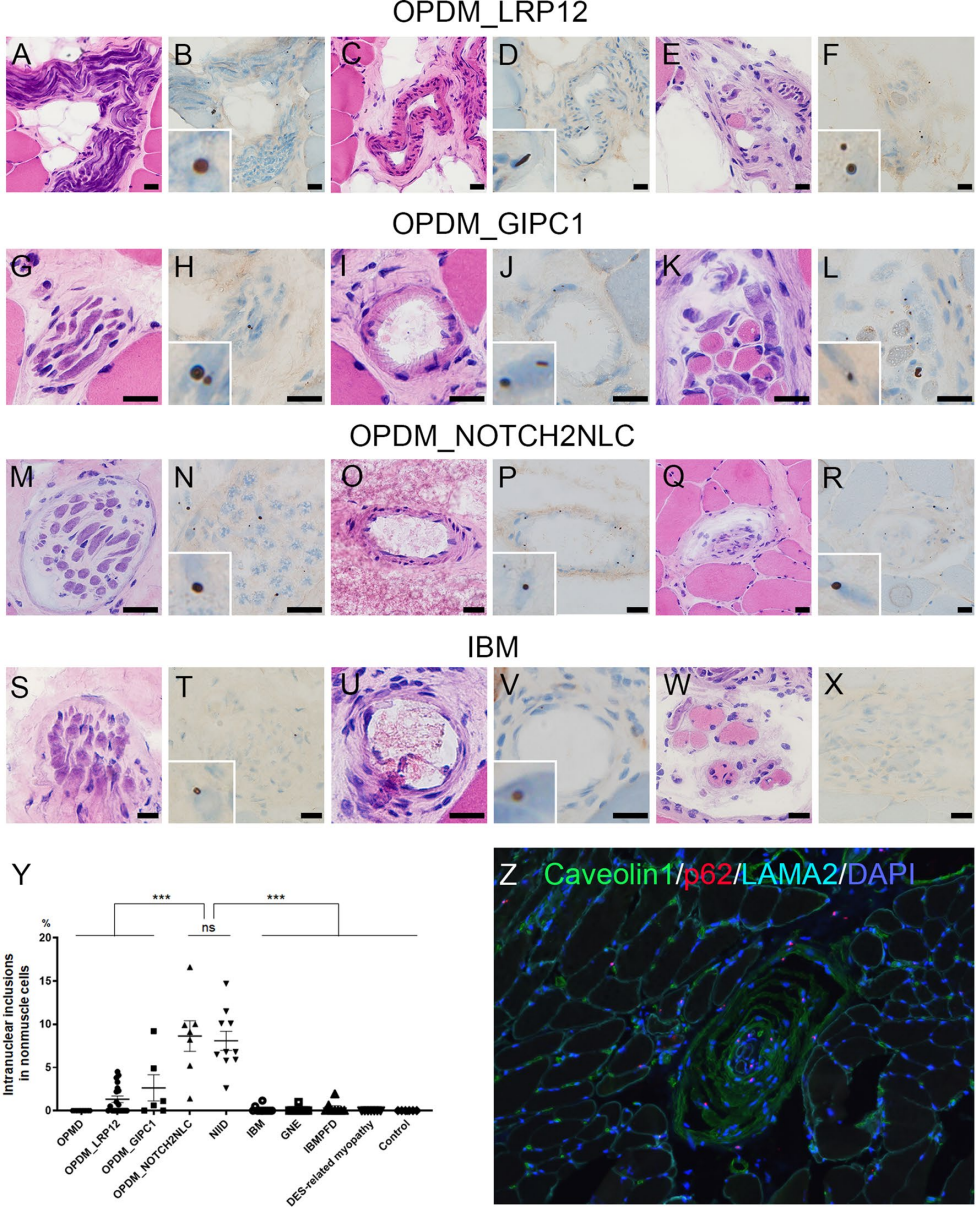
Intra-nuclear inclusions of non-muscle cells in muscle biopsy. H&E (**a**, **c**, **e**, **g**, **i**, **k**, **m**, **o**, **q**, **s**, **u**, **w**) and p62 (**b**, **d**, **f**, **h**, **j**, **l**, **n**, **p**, **r**, **t**, **v**, **x**) staining of serial sections of the muscle sample for each disease. The p62-positive INIs are observed in Schwann cells, pericytes, and/or perineurium cells (**b**, **h**, **n**, **t**), blood vessels (**d**, **j**, **p**, **v**), and muscle spindles (**f**, **l**, **r**). The p62-positive INIs are not seen in the muscle spindles from IBM (**w**, **x**). **a**–**x** All black bars show 20 µm. **y** Frequency of p62-positive INIs in non-muscle cells in total for each disease. Error bars indicate mean ± SEM. *, *p* < 0.05; ns, no significance. **z** The muscle spindle taken from OPDM_NOTCH2NLC shows p62-positive INIs (red color). *H&E* hematoxylin and eosin; *INIs* intra-nuclear inclusions; *IBM* inclusion body myositis; *OPDM* oculopharyngodistal myopathy; *NIID* neuronal intranuclear inclusion disease; *IBMPFD* inclusion body myopathy with Paget’s disease of bone and frontotemporal dementia; *OPMD* oculopharyngeal muscular dystrophy

We further evaluated non-muscle-INIs in the blood vessels, Schwann cells/pericytes, perineurium, and muscle spindles, except the intrafusal muscle fibers, in muscle biopsy samples. The samples from the patients with OPMD (*n* = 15), DES-related myopathy (*n* = 7), and control muscle samples (*n* = 6) did not show non-muscle-INIs (Table 1). In contrast, non-muscle-INIs were observed in patients with OPDM_LRP12 (53%—10/19), OPDM_GIPC1 (67%—4/6), OPDM_NOTCH2NLC (100%—7/7), NIID (100%—10/10), IBM (18%—2/11), GNE myopathy (10%—1/10), and IBMPFD (25%—2/8) (Fig. 2a-y). The frequency of non-muscle-INIs was significantly higher in OPDM_NOTCH2NLC (8.6 ± 1.8% [*n* = 7]) than in OPDM_LRP12 (1.3 ± 0.4% [*n* = 19], *p* < 0.0001), OPDM_GIPC1 (2.6 ± 1.5% [*n* = 6], *p* < 0.0001), IBM (0.1 ± 0.1% [*n* = 11], *p* < 0.0001), GNE myopathy (0.1 ± 0.1% [*n* = 10], *p* < 0.0001), and IBMPFD (0.3 ± 0.2% [*n* = 8], *p* < 0.0001) (Fig. 2y). There was no significant difference in the frequency of non-muscle-INIs among the patients with OPDM_GIPC1 and OPDM_LRP12, IBM, GNE myopathy, IBMPFD, and DES-related myopathy and the control samples.

## Discussion

On histopathology, OPDM and OPMD show considerably indistinguishable features, characterized by the presence of rimmed vacuoles and angular-shaped atrophic fibers [7, 11, 14]. Furthermore, both conditions present with adult-onset progressive ptosis, ophthalmoplegia, bulbar symptoms, and limb muscle weakness. Although distal limb muscles are predominantly affected in OPDM and proximal limb muscles in OPMD, some of the OPMD patients are known to clinically develop an OPDM phenotype, while some OPDM patients present predominantly proximal muscle involvement, making the diagnosis even more difficult without genetic information [3, 7, 8, 10].

In this study, the frequency of myo-INIs was found to be higher in OPMD (11.9 ± 1.1%, range 5.9–18.6%) than in the three types of OPDM (0.9–1.5% on average, range 0–2.8%, *p* < 0.0001). On the other hand, non-muscle-INIs were not seen in OPMD samples. Moreover, the non-muscle-INIs were seen in all of the patients with OPDM_NOTCH2NLC and NIID, and some with OPDM_GIPC1 and OPDM_LRP12. These results suggest that the high frequency of myo-INIs, together with the absence of non-muscle-INIs, could differentiate OPMD from OPDM in muscle biopsy pathology. Although patients with IBM, GNE myopathy, and IBMPFD also showed non-muscle-INIs, the frequency of positive nuclei was low (< 2%). This may indicate that the development of non-muscle-INIs is unlikely to be a mainstream pathologic process in these conditions.

PABPN1 accumulation was reported to be useful in differentiating OPMD from other myopathies including myotonic dystrophy, inflammatory myopathies, and muscular dystrophies [4]. The myo-INIs found in OPMD in our study may reflect the PABPN1 accumulation described in the previous report as PABPN1 was colocalized with p62 aggregates in the myonuclei on immunohistochemistry and also because such intra-myonuclear PABPN1 accumulation was absent in other vacuolar myopathies, including OPDM. However, it must be noted that the previous study did not evaluate PABPN1 accumulation in OPDM. We believe that the p62 expression in the myo-INIs is more useful in the diagnosis of OPMD as p62 immunohistochemistry is widely used to evaluate RVMs such as IBM, while PABPN1 immunohistochemistry is not. Considering that intranuclear PABPN1 accumulation is seen only in OPMD and that the size of tubulofilamentous inclusion is reported to be different between OPMD and OPDM [7, 10, 15], the two diseases may have a difference in the mechanism of their etiopathogenesis. Furthermore, the presence of p62-positive nuclear inclusion in non-muscle cells, which are absent in OPMD but present in OPDM, contributes to the diagnosis of OPDM and may also imply a difference in their pathomechanism.

## Conclusions

OPMD can be differentiated from other RVMs, including OPDM, by the frequent presence of p62-positive myonuclei (myo-INIs). Furthermore, the presence of p62 expression in non-muscle-INIs may facilitate the diagnosis of OPDM.

## Supplementary Information

**Additional file 1: Table S1.** Information about the detailed clinical findings in all patients.

## Data Availability

The data supporting the findings in this study are available from the corresponding author upon request.

## Abbreviations

OPDM: Oculopharyngodistal myopathy
LRP12: Low-density lipoprotein receptor-related protein 12
GIPC1: GIPC PDZ domain containing family member 1
NOTCH2NLC: Notch homolog 2 N-terminal-like protein C
RILPL1: Rab interacting lysosomal protein like 1
PABPN1: Poly-adenine-binding protein nuclear 1
INI: Intranuclear inclusion
NIID: Neuronal intranuclear inclusion disease
RVM: Rimmed vacuolar myopathy
NCNP: The National Center of Neurology and Psychiatry
IBM: Inclusion body myositis
IBMPFD: Inclusion body myopathy with Paget’s disease of bone and frontotemporal dementia
